# Management of Spinal Bone Metastases With Radiofrequency Ablation, Vertebral Reinforcement and Transpedicular Fixation: A Retrospective Single-Center Case Series

**DOI:** 10.3389/fonc.2021.818760

**Published:** 2022-01-21

**Authors:** Giuseppe Roberto Giammalva, Roberta Costanzo, Federica Paolini, Umberto Emanuele Benigno, Massimiliano Porzio, Lara Brunasso, Luigi Basile, Carlo Gulì, Maria Angela Pino, Rosa Maria Gerardi, Domenico Messina, Giuseppe Emmanuele Umana, Paolo Palmisciano, Gianluca Scalia, Francesca Graziano, Massimiliano Visocchi, Domenico Gerardo Iacopino, Rosario Maugeri

**Affiliations:** ^1^ Neurosurgical Clinic, AOUP “Paolo Giaccone”, Post Graduate Residency Program in Neurologic Surgery, Department of Biomedicine Neurosciences and Advanced Diagnostics, School of Medicine, University of Palermo, Palermo, Italy; ^2^ Department of Neurosurgery, Cannizzaro Hospital Trauma Center Gamma Knife Center, Catania, Italy; ^3^ Department of Neurosurgery, Highly Specialized Hospital and of National Importance “Garibaldi”, Catania, Italy; ^4^ Institute of Neurosurgery, Catholic University School of Medicine, Policlinico “A. Gemelli”, Rome, Italy

**Keywords:** RFA, PMMA, spinal metastases, spinal fixation, vertebral reinforcement

## Abstract

Spine is a frequent site of bone metastases, with a 8.5 months median survival time after diagnosis. In most cases treatment is only palliative. Several advanced techniques can ensure a better Quality of Life (QoL) and increase life expectancy. Radiofrequency ablation (RFA) uses alternating current to produce local heating and necrosis of the spinal lesion, preserving the healthy bone. RFA is supported by vertebral reinforcement through kyphoplasty and vertebroplasty in order to stabilize the fracture with polymethylmethacrylate (PMMA) injection, restoring vertebral body height and reducing the weakness of healthy bone. The aim of this study is to demonstrate the efficacy and advantages of RFA plus vertebral reinforcement through PMMA vertebroplasty and fixation in patients affected by bone spinal metastases. We retrospectively analyzed 54 patients with thoraco-lumbar metastatic vertebral fractures admitted to our Unit between January 2014 and June 2020. Each patient underwent RFA followed by PMMA vertebroplasty and transpedicle fixation. We evaluated pain relief through the Visual Analogue Scale (VAS) Score and PMMA vertebral filling based on the mean Saliou filling score. Analysis of variance (ANOVA) was used to test pain relief with statistical significance for p<0.05. A total of 54 patients (median age 63,44 years; range 34-86 years), with a total of 63 infiltrated vertebrae, were treated with RFA, PMMA vertebroplasty and transpedicular screw fixation; average operative time was 60.4 min (range 51–72). The preoperative average VAS score decreased significantly from 7.81 to 2.50 (p < 0.05) after 12 months. Based on Saliou filling score, filling was satisfactory (12–18) in 20 vertebrae (31,7%), mediocre (6–11) in 33 vertebrae (52,4%), inadequate (0–5) in 10 vertebrae (15,9%). A consistent PMMA filling of vertebral bodies was successfully achieved with significant pain relief. Concomitant RFA, PMMA vertebroplasty and pedicle screw fixation represent a safe and effective technique for the management of spinal metastases, improving clinical outcome and pain control.

## Introduction

Spine is a frequent site of bone metastases. The high vascularity related to the Batson’s venous plexus and arterial spreads may contribute to metastatic localization (1, 2). In US, this is the second cause of death and the median overall survival after surgery is 8.5 months in patients with colon, breast, prostate, thyroid, renal cell, lung, and skin cancers. Until recent times, palliation is the aim of most treatments; nevertheless, several advanced techniques can ensure a better quality of life and increase life expectancy in patients with spinal metastases (3–7).

Radiofrequency ablation (RFA) uses alternating current to produce local heating and necrosis of the spinal lesion, while preserving the healthy bone. In 1931, RFA was used for the first time by Krischner to treat trigeminal neuralgia through thermocoagulation of gasserian ganglion. In late 1950s, Cosman and Aronow fine-tuned the first radiofrequency machine. Finally, RFA has been used by Rosenthal since 1992 as palliative treatment of bone metastases (8–11).

In several cases of metastatic lesion, pain can be caused by a combination of fractures and related vertebral instability, periosteal involvement, and pro-inflammatory cytokines, and pain relief is probably achieved by reduction of osteoclast activity and destruction of periosteal nerve endings (7, 12–15).

Percutaneous techniques, such as vertebroplasty, kyphoplasty and RFA achieve a key role in management of vertebral metastatic lesions (16–20).

This technique has been already performed to treat colon cancer, liver, and kidney tumors. However, vertebral RFA alone may increase the risk of fracture since it can weaken healthy bone. For this reason, this technique is usually supported by vertebral reinforcement through kyphoplasty and vertebroplasty, in order to stabilize the fracture with injection of bone cement (i.e. polymethylmethacrylate, PMMA), thus restoring vertebral height (1, 7, 21–23). The aim of this study is to demonstrate the efficacy and advantages of RFA plus vertebral fixation and a reinforcement through PMMA vertebroplasty in patients affected by spinal metastases and to demonstrate the relationship between vertebral restoration and pain relief.

## Methods

From January 2014 to June 2020, a total of 54 patients (29 Males and 25 females) affected by thoraco-lumbar vertebral body metastases were admitted to the Department of Neurosurgery, BIND, University of Palermo (Italy) and they were retrospectively analyzed. All the patients underwent RFA plus vertebral body reinforcement for the treatment of vertebral metastases. In addition, pedicle screw fixation of the adjacent vertebrae was performed to restore stability.

Inclusion criteria were: Karnofsky score >= 60, unremitting thoraco-lumbar pain (VAS score >=5), osteolytic lesion on neuroimaging, unresecable tumors (according to Tokuhashi score), intractable pain with chemotherapy, radiation therapy and refractory to analgesic drugs. Exclusion criteria were: Karnofsky score <60, mild thoraco-lumbar pain (VAS <5), osteoblastic tumors on neuroimaging, general contraindications for surgery (infection, allergy, bleeding diseases), intradural and intramedullary tumors and neurological impairments caused by spinal metastasis itself. Patients’ demographics and relative comorbidities are summarized in **Table 1**.

**Table 1:** Demographic data of patients selected for this clinical series.

Patient	Age	Gender	Primary cancer	Localization	Comorbidities	Pre Op VAS	Post Op 1 weekVAS	Post Op 1 monthVAS	Post Op3 monthsVAS	Post Op 6 monthsVAS	Analgesic reduction	Sanliou filling score
1	67	M	Lung	D12	Hypertension, diabetes	8	6	4	2	2	YES	12
2	81	F	Kidney	L4	Diabetes	7	6	3	1	1	YES	11
3	77	F	Breast	D10,D11,D12	Pneumonia	9	7	5	4	2	YES	11,8,13
4	56	M	Melanoma	D8	Smoker, intestinal polyposis	7	6	4	4	1	NO	5
5	65	F	Spinalioma	D7-D8	Angina pectoris	9	7	7	3	2	NO	9
6	81	F	Liver	L3-L5	Hypertension, Diabetes	7	5	4	2	1	YES	7,8
7	59	M	Kidney	D10-D11	Dyslipidemia	9	7	4	4	2	NO	4
8	70	M	Bladder- Prostate	L1	Crohn’s disease	7	7	4	3	1	YES	11
9	80	F	Breast	D9,D10,D11	Hypertension, smoker	7	6	6	4	2	YES	12,9,7
10	82	F	Breast	D7,D8	Angina pectoris	8	5	5	2	2	YES	9
11	80	F	Liver	D7	Dyslipidemia, asthma	8	6	4	4	1	NO	5
12	86	M	Prostate	D8	Stroke, amyloid angiopathy	9	7	5	4	3	YES	15
13	77	M	Lung	D8	Hypertension, gastric ulcer	6	5	5	4	4	YES	11
14	75	M	Lung	D10	COPD	7	5	4	3	3	NO	7
15	61	F	Lung	L1	Hypertension, asthma	7	6	5	4	3	YES	15
16	61	F	Breast	D11	COPD	8	6	5	3	3	YES	11
17	42	F	Breast	D4,D11,D12	Congestive heart failure	8	7	6	4	3	YES	9
18	67	M	Lung	D4	Gastric ulcer, smoker, dyslipidemia, heart attack	7	5	4	4	2	YES	13
19	75	M	Liver	D9	COPD, hypertension	9	6	4	4	1	YES	11
20	47	F	Breast	D12-L1	Smoker, migraine	8	6	6	3	2	YES	16
21	85	M	Kidney	L3	Smoker, pneumonia	9	7	6	6	3	NO	7
22	51	F	Breast	D9-D10	hypertension	8	7	5	4	3	YES	11,8
23	72	M	Kidney	D10	Smoker, asthma, pneumonia	7	6	4	3	2	NO	8
24	62	M	Sarcoma	D9	Smoker	7	5	5	3	2	YES	13
25	53	F	Breast	D5	Hypertension, smoker	9	8	6	6	3	YES	12
26	73	F	Lung	D8,D9,D11	Diabetes	10	8	8	7	4	NO	8,5,7
27	72	M	Lung	L3	Smoker, pneumonia, glaucoma	7	4	3	3	1	YES	11
28	57	F	Leiomiosarcoma	L2	COPD, peripheral arterial disease	7	5	5	5	2	NO	9
29	72	F	Cordoma	D6	Right bundle branch block, asthma	8	4	4	4	3	NO	5
30	50	F	Mieloma	L5	smoker	9	7	6	6	4	NO	6
31	56	M	Breast	D7	Asthma, diverticulosis	7	6	5	3	2	NO	5
32	71	F	Lung	D6	smoker	6	5	3	3	2	YES	13
33	58	M	Mieloma	D7,D8	Hypertension, smoker	7	5	3	3	2	YES	12,10
34	69	M	Lung	D5,D6	Unruptured aneurysm	6	5	3	3	2	YES	7
35	71	M	Prostate	D8	Diabetes	7	6	5	4	3	YES	14
36	62	M	Lung	L3	Asthma	8	7	6	4	2	YES	11
37	54	M	Lung	L2	Smoker	7	2	2	3	4	YES	10
38	39	F	Breast	D10	/	7	2	2	3	2	YES	12
39	57	M	Bladder	L4	smoker	9	5	3	3	4	YES	4
40	63	M	Bladder	D12	smoker	7	2	2	2	4	YES	14
41	62	F	Breast	D12	hypertension	7	5	3	3	3	NO	9
42	69	M	Lung	L1	Hypertension, diabetes	8	4	5	3	3	NO	8
43	60	M	Lung	L3	COPD	10	3	3	3	3	YES	12
44	34	F	Breast	L5	Angina pectoris	8	5	4	3	3	NO	10
45	53	M	Lung	L1	Diabetes, hypertension	9	3	3	3	3	YES	5
46	49	F	Kidney	L2	smoker	7	5	3	2	2	NO	13
47	57	M	Breast	D12	smoker	6	4	2	2	3	YES	9
48	58	M	Lung	L1	Hypertension	8	3	2	2	2	NO	5
49	67	M	Lung	D11	hypertension	10	3	4	4	4	YES	12
50	69	F	Breast	L3	Pneumonia	7	2	2	3	4	YES	14
51	58	F	Kidney	L4	Glaucoma, unruptured aneurysm	8	6	3	3	2	YES	12
52	62	M	Breast	D10	Asthma	10	4	2	2	4	YES	9
53	39	F	Lung	L2	Smoker	8	2	2	2	2	NO	13
54	53	M	Melanoma	D8	Smoker	9	3	4	2	2	YES	8

Before the procedure, written informed consent was obtained from all patients. Each patient underwent radiofrequency ablation followed by PMMA vertebroplasty and subsequent open or percutaneous transpedicle screw fixation with or without posterior decompression during the same surgical procedure. RFA was performed with the STAR Tumor Ablation System (consisting of the SpineSTAR ablation instrument and the MetaSTAR generator; DFine, San Jose, CA, USA). The SpineSTAR is an articulated navigational bipolar radiofrequency electrode containing a pair of thermocouples positioned along the length of the electrode, 10 and 15 mm from the center of the ablation zone. This area is reached *via* a transpedicular approach, in order to avoid vascular injuries. The ablation zone has 3: 2 length-to-width aspect ratio, with a maximum ablated zone of 3 cm × 2 cm when the proximal thermocouple reaches 50 degrees Celsius. This system has been used to treat pedicles and anterior/posterior vertebral bodies metastasis especially thanks to the STAR probe that can be curved to reach different vertebral portions through the same entry point. The MetaSTAR generator continuously displays the temperature from the two thermocouples, in order to allow real-time monitoring of the peripheral edge of the ablation zone. After RFA, cement reinforcement (StabiliT Vertebral Augmentation System; DFine) was performed in all cases through the same working cannula. The entire procedure was performed under fluoroscopic guidance to evaluate cement distribution and screw trajectories.

In the first post-operative day, each patient performed a thin-slice CT scan. PMMA vertebral filling was evaluated using the mean Saliou filling score. Each vertebral body was divided into 18 3D equal portions (nine portions in AP projection, nine portions in LL projection). Each portion was considered filled if cement was visible inside. Filling score ranged from one to eighteen. According to Saliou et al., we considered the filling satisfactory if the score was more than twelve, mediocre when the score ranged from six to twelve, inadequate if the score was less than six (24).

We evaluated use of analgesic drugs (NSAIDS, Steroids, Opioids) and pain relief through Visual Analogic Scale score (0 to 10 ranged). The questionnaire was administered before surgery, one week, one, three, six months after surgical procedure.

## Results

**Figure 1:**
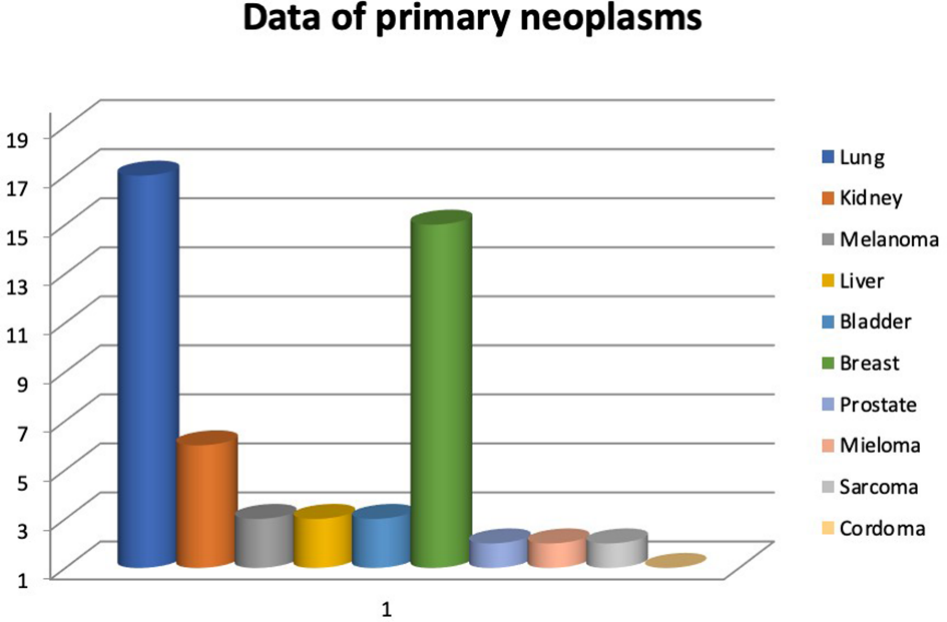
Frequency of primary neoplasm.

**Figure 2:**
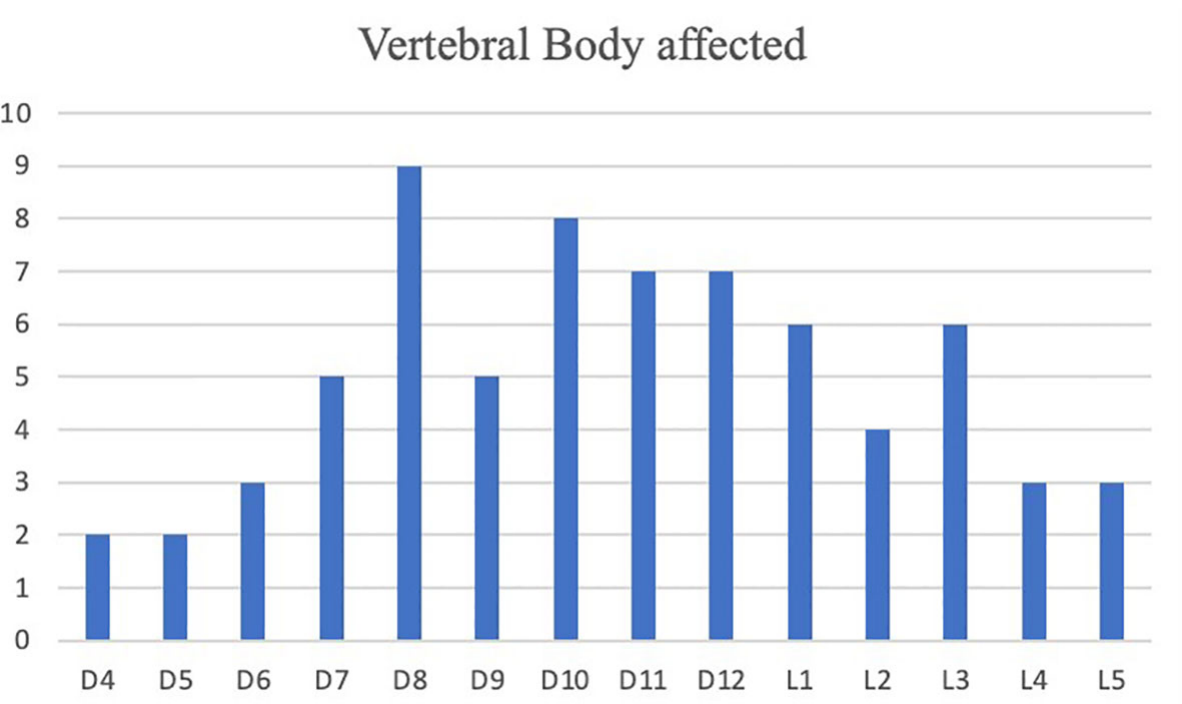
Frequency of neoplastic vertebrae treated by radiofrequency ablation and vertebroplasty.

During the abovementioned time frame, 54 patients for a total of 63 vertebrae were treated. Median age was 63,44 years (range 34-86 years). The primary neoplasms sites associated to vertebral metastasis in order by frequency were lung, breast, kidney, melanoma, liver, bladder, prostate, multiple myeloma, sarcoma, chordoma **(** **Figure 1** **)**. Levels of metastatic vertebrae were showed in **Figure 2**. The combination of radiofrequency ablation followed by vertebroplasty and subsequent open or percutaneous transpedicular screw fixation and posterior decompression was performed in 23 out of 54 patients (42.6%).

The most common patients’ comorbidities are hypertension (14%), diabetes (12,72%), smoking (34,54%), asthma(12,72%), pneumonia (9,09%), glaucoma (3,63%), Crohn’s disease (1,81%), abdominal unruptured aneurysm, Chronic obstructive pulmonary disease (COPD) (9,09%), gastric ulcer (3,63%), dyslipidemia (5,45%), cardiovascular disorders (12,72%), bowel disease (3,63%), amyloid angiopathy (1,81%), stroke (1,81%), migraine (1,81%). (**Table 1**)

**Figure 3:**
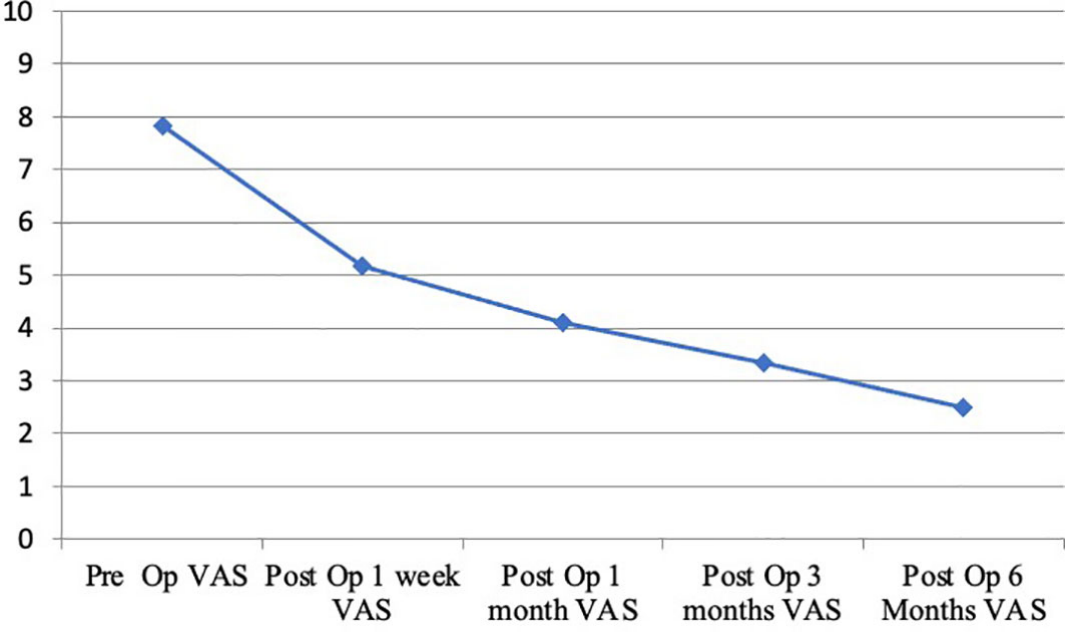
Trend of the visual analogue scale (VAS, 0 to 10 ranged) in patients with back pain during a six month follow-up.

**Figure 4:**
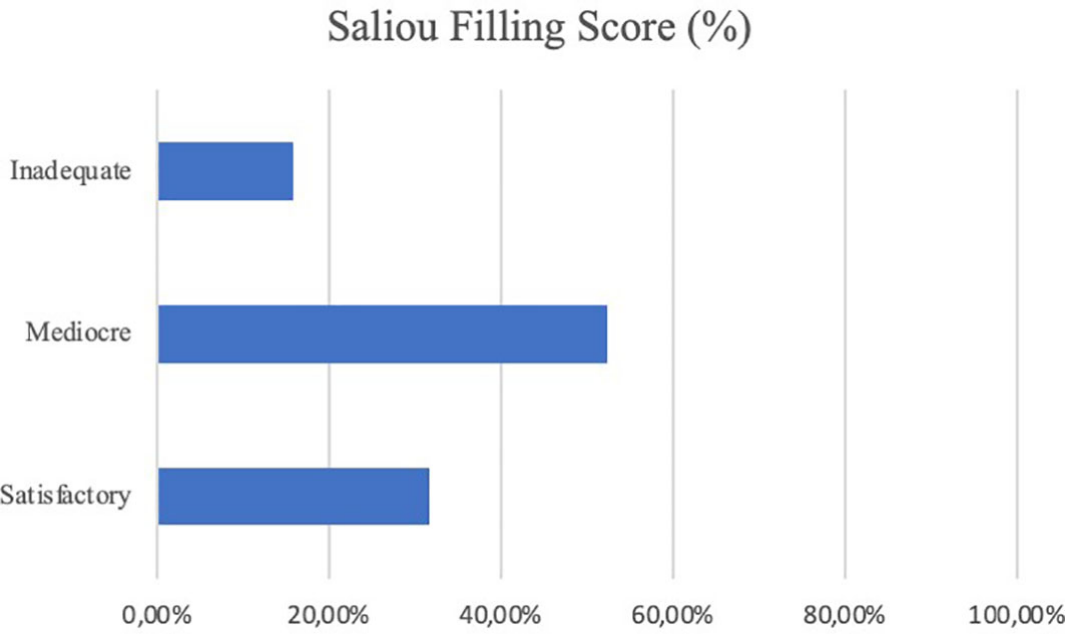
Filling grade of treated vertebrae according to Saliou filling score (percentage of treated vertebrae).

Surgical treatment had a significant impact on pain relief. Mean preoperative VAS score was 7.81/10 (range 6-10). A significant reduction of overall subjective pain perception was registered, with a persistent reduction in VAS score 1 week (5,16/10), 1 month (4,11/10), 3 months (3,35/10) and 6 months (2,50/10) respective after surgery. **(** **Figure 3** **)** These results, compared to preoperative data, suggest that our combined treatment had a significant impact on pain relief (p < 0.05), and in QoL as consequence. Intraoperative neuromonitoring (IONM) was performed during the entire procedure in order to avoid neurological complications. PMMA injection within the ablated cavity was monitored by continuous fluoroscopic guidance. After surgery, a thin-slice CT scan was performed, in order to evaluate cement distribution. According to the previously described radiological evaluation, Saliou filling score was satisfactory (12–18) in 20 vertebrae (31,7%), mediocre (6–11) in 33 vertebrae (52,4%), inadequate (0–5) in 10 vertebrae (15,9%). **(** **Figure 4** **)**

A small amount of cement leakage into perivertebral tissues was found in 7 vertebrae (11,1%). However, no neurovascular structure was affected and no vascular or neurological injuries were recorded. Peri-operative mortality related to the surgical procedure was 0%.

### Case Illustration

**Figure 5:**
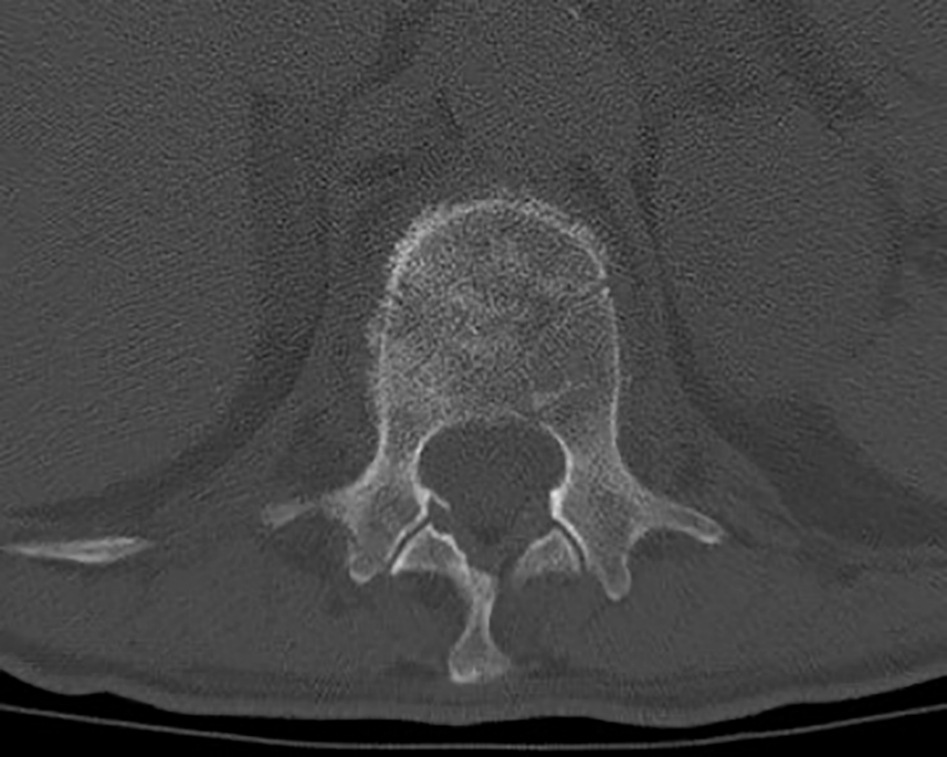
Axial thoraco-lumbar CT scan showing L1 metastatic lesion with altered bone density and osteolytic areas.

**Figure 6:**
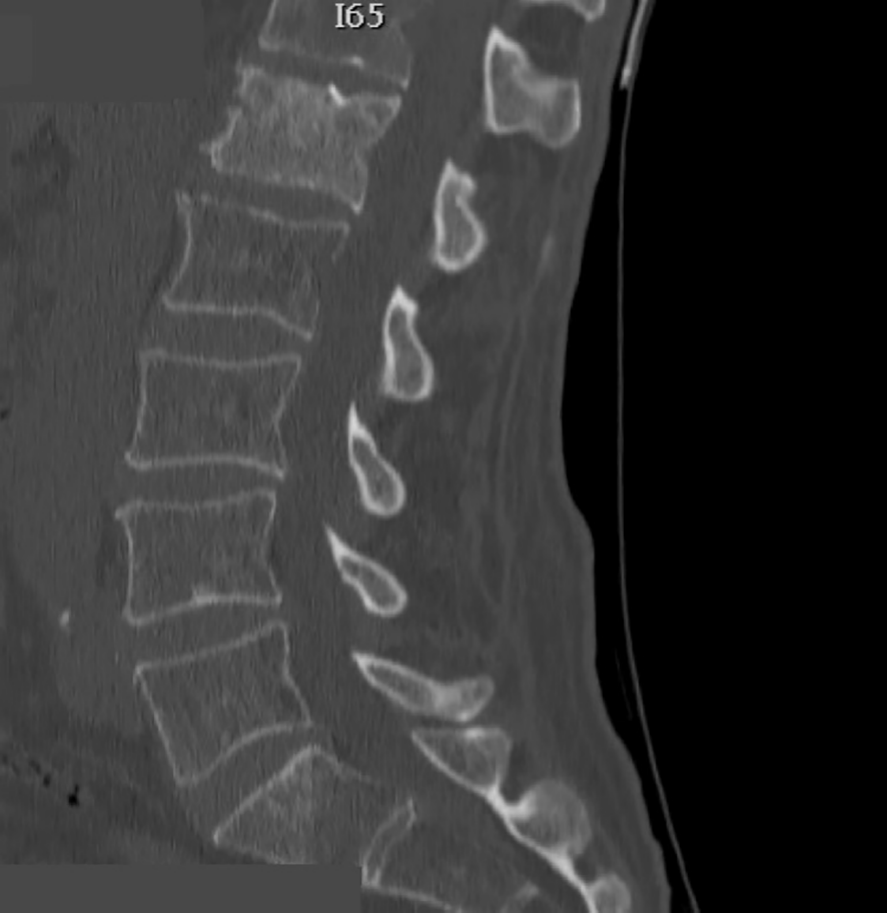
Sagittal thoraco-lumbar CT scan showing L1 metastatic lesion with altered bone density and osteolytic areas.

**Figure 7:**
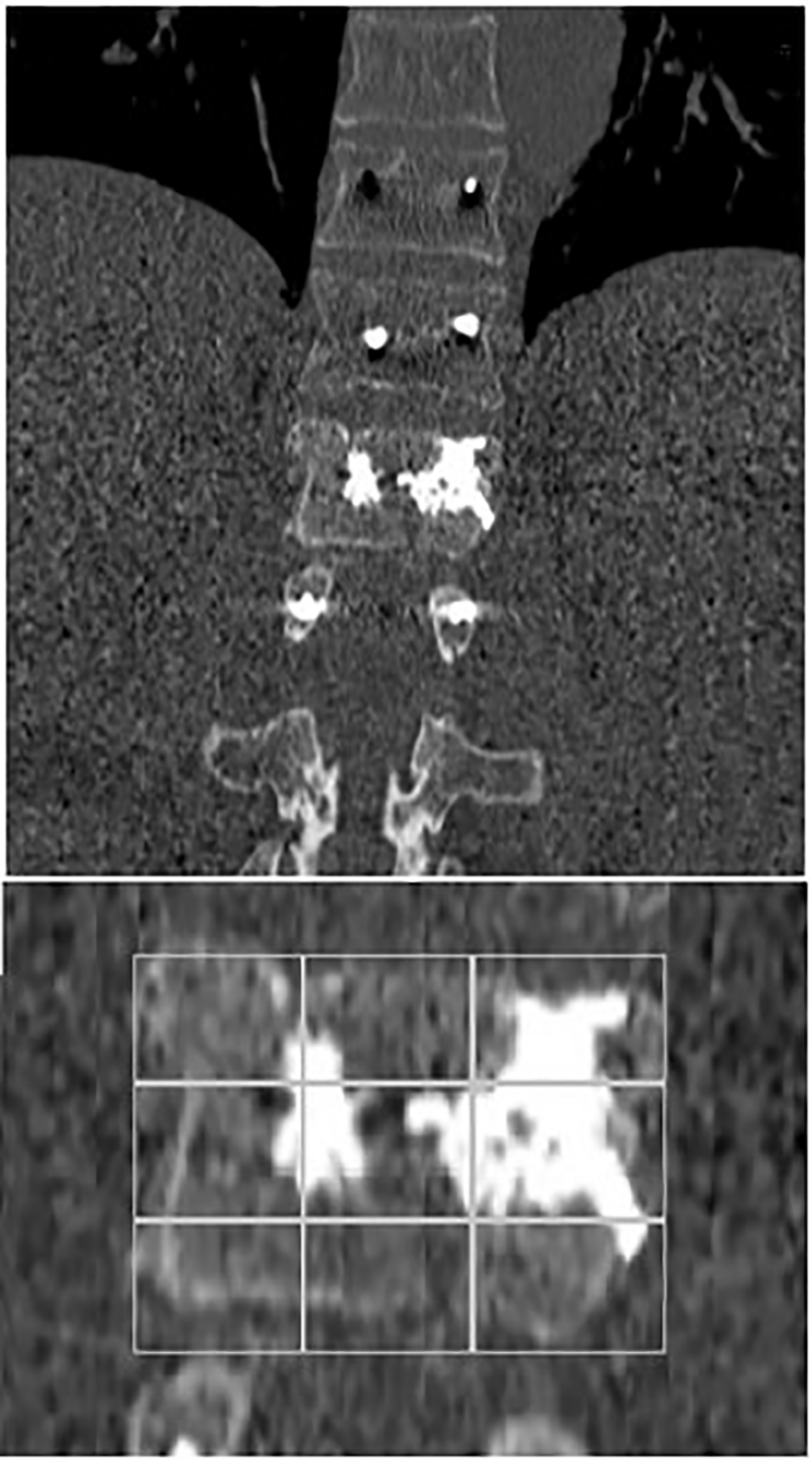
Coronal early post-operative CT scan showing an L1 average cement filling based on Saliou filling score 11/18 (6 + 5) and a T11-T12-L2 vertebral fixation.

**Figure 8:**
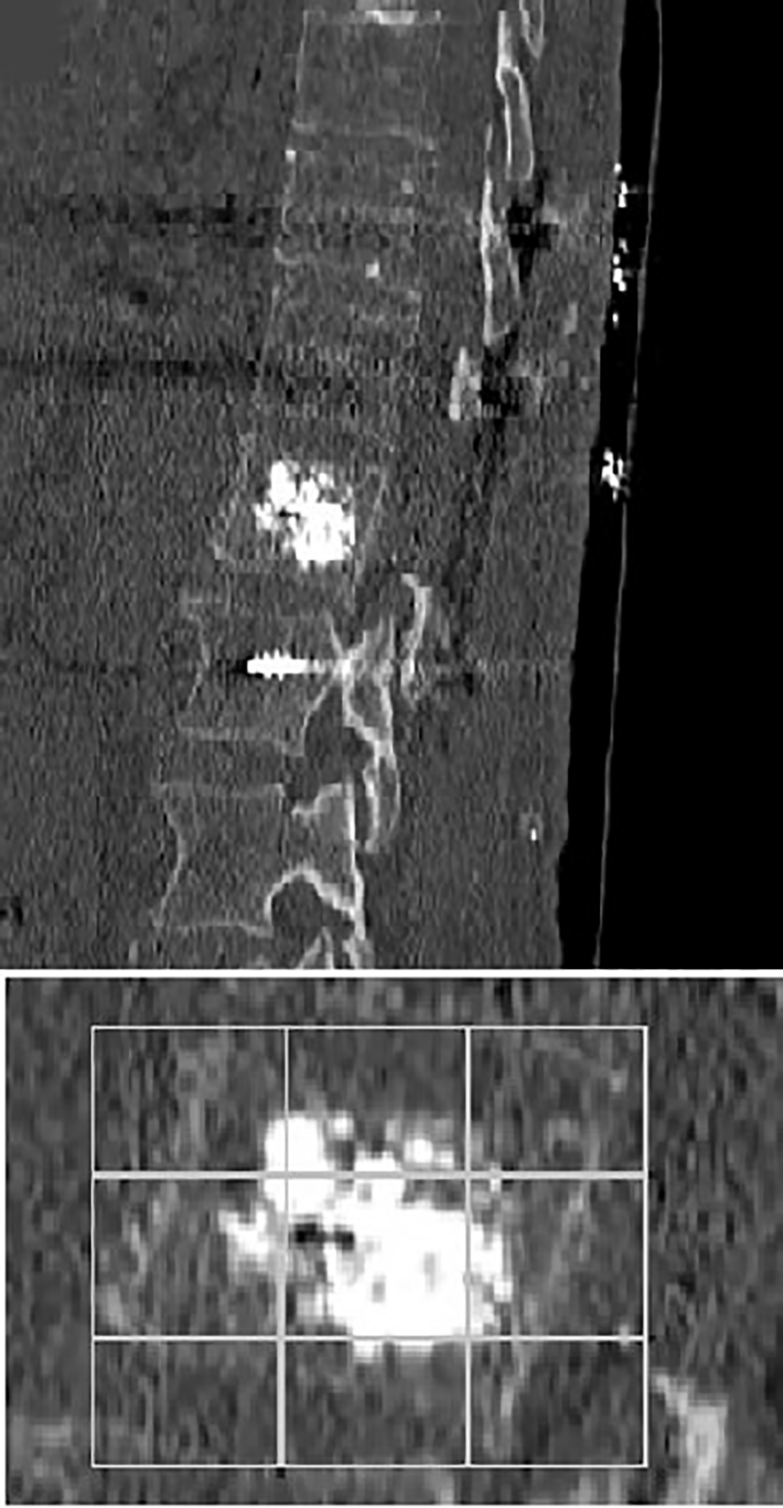
Sagittal early post-operative CT scan showing an L1 average cement filling based on Saliou filling score 11/18 (6 + 5) and a T11-T12-L2 vertebral fixation.

A 70-year-old male patient with a previous history of a prostatic carcinoma, was admitted to our department complaining intense low back pain (VAS score 7/10), which worsened two weeks before admission. A lumbar CT scan showed an osteolytic L1 vertebral body lesion. (**Figures 5**, **6**) Therefore, he underwent surgery with L1 laminectomy and neural decompression, biopsy, RFA of L1 lesion with STAR Tumor Ablation System (DFine, San Jose, CA, USA) and T11-T12-L2 posterior thoraco-lumbar fixation. The post-operative course was uneventful, with a significant pain relief (VAS 7/10 1 week after procedure, 4/10 after 1 month, 3/10 after three months, 1/10 after six months). A post-operative thoracolumbar CT scan showed an average cement filling (11 Based on Saliou filling score) and correct screw placement. **(** **Figures 7**, **8**).

## Discussion

RFA is currently used as one of the most safe and effective technique for bone metastases. This technique, based on the use of alternating current with a bipolar or monopolar device, provokes a coagulative necrosis of the tumoral lesion. Nevertheless, as a main consequence, this technique can weaken surrounding healthy bone. For this reason, the concomitant use of Polymethylmethacrylate (PMMA) guarantees a good result in terms of pain and stability. PMMA is placed with a needle in the affected vertebra and it acts as a “glue”: it fills the vertebra and then it is fixed through an exothermic reaction (25–27).

Pain relief is obtained through the techniques previously mentioned, with a lesser use of painkillers during follow up. Nerve fibers transmission, tumor cells and their microenvironment enriched by proinflammatory cytokines, osteoclast activity may together participate to the pathophysiology of metastatic pain. Since RFA interferes with these mechanisms, it has a positive effect on pain. In our cohort we obtained a harmless ablation through the use of an articulated electrode with two thermocouples and a continuous monitoring of local temperature and tissue impedance. In particular, mean VAS scores were at baseline (7,81/10), at 1 week (5,16/10), and at months 1(4,11/10), 3 (3,35/10), and 6 months (2,50/10) respectively; with an optimum decrease in post-operative course. As regards recent literature, our series was compared to the largest case series in order to evaluate the positive impact of RFA in relieving pain. (**Table 2**).

**Table 2:** Literature review of the largest reported clinical series of patients treated by RFA and PMMA vertebral reinforcement.

References	Mean Procedure Time (minutes)	Mean Ablation Temperature (°C)	Treatment	Number of Patients	Number of Vertebrae	Number of Complications	Preoperative Pain	Postoperative Pain
Toyota et al., 2005 (28)	Radiofrequency ablation 4.1	71.8	RFA+PMMA	17	23	2	6.3	Not reported
Hoffman et al., 2008 (29)	Not reported	95	RFA+PMMA	22	28	10	8.5	5.5
Munk et al., 2009 (30)	Radiofrequency ablation 9.1	Not reported	RFA+PMMA	19	25	7	7.9 +/- 1	Not reported
Sandri et al., 2010 (20)	53	Not reported	RFA+PMMA	11	11	0	8	1.9
Lane et al., 2011 (31)	Radiofrequency ablation 12	95	RFA+PMMA	36	53	21	7.2 +/- 1.69	Not reported
Clarencon et al., 2013 (32)	Not reported	Not reported	RFA+PMMA	12	12	3	6.4 +/- 2.7	1.9 +/- 2.4
Burgard et al., 2014 (33),	Not reported	95	+/-RFA+PMMA	29	26	6	Not reported	Not reported
Zheng et al., 2014 (34)	47.77 +/- 7.13	95 +/- 5	RFA+PMMA	26	38	0	7.69 +/- 1.12	6.62 +/- 1.02
Anchala et al, 2014 (35)	6,02 (radiofrequency ablation)	50	RFA	92	128	Not reported	7.51	2.25
Wallace et al., 2015 (36)	Radiofrequency ablation 8.32	Not reported	RFA+PMMA	105	105	4	8.0	3.9
Madaelil et al., 2016 (37)	8.6 +/- 3.4	Not reported	RFA +/- PMMA	11	16	0	8	Not reported
Reyes et al., 2017 (38)	Not reported	Not reported	RFA+PMMA	49	72	0	7.9 +/- 2.5	3.5 +/- 2.6
Maugeri et al., 2017 (19)	Not reported	50	RFA+PMMA	18	18	0	8.05	3.0
Sayed et al., 2019 (1)	9.56 per level	Not reported	RFA+PMMA	30	34	0	5.77 +/- 2.81	2.61+/- 2.28
Present study, 2021	60.4	50	RFA+PMMA+FIXATION	54	63	0	7.8	2.5

Indeed, Sandri et al. in their systematic review reported that 11 patients treated with RFA and vertebroplasty presented a significant pain relief (1, 20). Furthermore, in 2014, Anchala et al. reported a remarkable VAS score improvement in 92 patients with a total of 128 lesions treated using RFA with or without vertebral cement reinforcement (1, 35, 39). Schaefer et al. showed a case of a patient with an osteolytic lesion at L3 who was treated successfully with RFA and vertebroplasty in one step (19, 40–43).

Moreover, PMMA neurotoxicity could contribute to RFA painkiller effect since it could destroy nerves terminations and it gives a better stability to the spinal column at the same time (19, 20, 34, 44–46). As regard vertebral stability, our study analyzed PMMA filling of affected vertebrae based on Saliou filling score. Filling was considered satisfactory if the score was more than 12 (2/3 of the vertebra), mediocre when the score ranged from 6 to 12, and inadequate when the score was less than 6. After surgery, in order to evaluate if the procedure was performed correctly and with the proper amount of PMMA, a thin-slice CT scan was mandatory. Based on Saliou filling score was satisfactory (12–18) in 20 vertebrae (31,7%), mediocre (6–11) in 33 vertebrae (52,4%), inadequate (0–5) in 10 vertebrae (15,9%).

To our knowledge this is the first reported experience on combined RFA and vertebral reinforcement followed by transpedicular screw fixation. Cianfoni et al. reported a percutaneous technique called SAIF (stent screw–assisted internal fixation) following RFA and vertebral reinforcement that, through a minimal invasive procedure, ensures stability and height restoration in pathological and osteoporotic vertebral bodies. This is the unique study provided by the literature review that looks up to our same goal. Nevertheless, although this is an interesting and innovative procedure, transpedicular screw fixation represents a faster and more accessible technique to reach the same results (47–49).

In our single center experience, we noticed that, according to literature data about RFA plus vertebral reinforcement (**Table 2**), these three combined approaches guarantee a significant and more noticeable pain relief thus representing a good therapeutic compromise for patients’ treatment with metastatic spinal lesions. Moreover, a transpedicular screw fixation can be significant useful when spinal instability, as the patients included in our study, occurs (according to the spinal instability neoplastic score), guarantying not only a considerable pain relief but also a vertebral body reconstruction and subsequent vertebral stability.

This study has certainly some limitations. This is a retrospective analysis of a single medical center, without control group. Nevertheless, it shows an heterogenous group of patients with different vertebral locations and different clinic features. Further prospective studies with larger patient’s cohorts are mandatory to better evaluate the specific role of RFA procedure combined with vertebroplasty and followed by spinal fixation in the management of vertebral metastatic disease.

## Conclusion

The combination of RFA, PMMA vertebroplasty and transpedicle screw fixation represents a safe and effective technique for the management of spinal metastases guarantying a good and long-lasting clinical outcome in terms of pain relief.

## Data Availability Statement

The original contributions presented in the study are included in the article. Further inquiries can be directed to the corresponding author.

## Ethics Statement

Ethical review and approval was not required for the study on human participants in accordance with the local legislation and institutional requirements. The patients/participants provided their written informed consent to participate in this study.

## Author Contributions

Conceptualization, GG, RC, and RM. Methodology, GG. Investigation, RC. Resources, UB, MP, and FP. Data curation, LBr, LBa, and CG. Writing—original draft preparation, RC and MAP. Writing—review and editing, GG, PP, and GS. Visualization, GU, DM, FG, and RG. Supervision, DI, MM, and RM. Project administration, DI. All authors have read and agreed to the published version of the manuscript. 

## Conflict of Interest

The authors declare that the research was conducted in the absence of any commercial or financial relationships that could be construed as a potential conflict of interest.

## Publisher’s Note

All claims expressed in this article are solely those of the authors and do not necessarily represent those of their affiliated organizations, or those of the publisher, the editors and the reviewers. Any product that may be evaluated in this article, or claim that may be made by its manufacturer, is not guaranteed or endorsed by the publisher.
